# Naturally Occurring and Experimentally Induced Rhesus Macaque Models for Polycystic Ovary Syndrome: Translational Gateways to Clinical Application

**DOI:** 10.3390/medsci7120107

**Published:** 2019-11-27

**Authors:** David H. Abbott, Jeffrey Rogers, Daniel A. Dumesic, Jon E. Levine

**Affiliations:** 1 Department of Obstetrics and Gynecology, Wisconsin National Primate Research Center, University of Wisconsin, Madison, WI 53715, USA; 2 Department of Molecular and Human Genetics and Human Genome Sequencing Center, Baylor College of Medicine, Houston, TX 77030, USA; jr13@bcm.edu; 3 Department of Obstetrics and Gynecology, David Geffen School of Medicine, University of California Los Angeles, Los Angeles, CA 90095, USA; DDumesic@mednet.ucla.edu; 4 Department of Neuroscience, Wisconsin National Primate Research Center, University of Wisconsin, Madison, WI 53715, USA; levine@primate.wisc.edu

**Keywords:** developmental programming, androgen excess, infertility, adipogenic constraint, insulin resistance, transgenerational transmission, testosterone-associated traits

## Abstract

Indian rhesus macaque nonhuman primate models for polycystic ovary syndrome (PCOS) implicate both female hyperandrogenism and developmental molecular origins as core components of PCOS etiopathogenesis. Establishing and exploiting macaque models for translational impact into the clinic, however, has required multi-year, integrated basic-clinical science collaborations. Paradigm shifting insight has accrued from such concerted investment, leading to novel mechanistic understanding of PCOS, including hyperandrogenic fetal and peripubertal origins, epigenetic programming, altered neural function, defective oocytes and embryos, adipogenic constraint enhancing progression to insulin resistance, pancreatic decompensation and type 2 diabetes, together with placental compromise, all contributing to transgenerational transmission of traits likely to manifest in adult PCOS phenotypes. Our recent demonstration of PCOS-related traits in naturally hyperandrogenic (High T) female macaques additionally creates opportunities to employ whole genome sequencing to enable exploration of gene variants within human PCOS candidate genes contributing to PCOS-related traits in macaque models. This review will therefore consider Indian macaque model contributions to various aspects of PCOS-related pathophysiology, as well as the benefits of using macaque models with compellingly close homologies to the human genome, phenotype, development and aging.

## 1. Introduction

Polycystic ovary syndrome (PCOS) bestows detrimental life-long consequences on a woman’s health and wellbeing [1,2]. Elevated testosterone (T) levels are the most common endocrinopathy [3]. Newly published International Guidelines for the Assessment and Management of PCOS [1] support a clinical diagnosis requiring at least two out of the following three (Rotterdam) criteria: high circulating T levels or excessive body hair, intermittent or absent menstrual cycles, and polycystic ovaries. Related, but distinctly different endocrine disorders, must also be excluded [3]. Employing Rotterdam PCOS diagnostic criteria reveals prevalence rates as high as 21% across a variety of human populations [4,5,6], and significant morbidity derives from known associations with type 2 diabetes (T2D), cardiovascular dysfunction, obesity, infertility and cancer [2,6,7]. PCOS thus places heavy burdens on health-care resources [8] that may exceed $14 billion annual costs in the US alone.

Rotterdam criteria recognize four PCOS phenotypes: classic PCOS with (type A) and without (type B) polycystic ovaries, ovulatory PCOS (type C) without intermittent or absent cycles, and “non-hyperandrogenic” PCOS (type D) without high T or excessive body hair [1,3]. Phenotype B, however, will likely be subsumed within Type A when three-dimensional ovarian ultrasonography and standardized AMH immunoassays become widely available enabling accurate characterization of polycystic ovaries [9]. So, in essence, PCOS comprises three distinct phenotypes. Prevalence of individual PCOS phenotypes, however, depends on the populations studied (Table 1). The majority of PCOS subjects derived from clinical referrals exhibit classic PCOS phenotypes (types A and B) [10]. In contrast, the majority of PCOS subjects recruited from local or unselected human populations exhibit the less severe ovulatory or “non-hyperandrogenic” phenotypes (types C and D, respectively) [10]. Such distinctions become important when examining PCOS etiopathogenesis since the consequences of pronounced cardiometabolic disease in clinical referral studies may intertwine PCOS consequence with cause, thus providing less than ideal targets to emulate in animal models seeking foundational mechanisms for novel therapies. Not surprisingly, PCOS and its accompanying morbidities remain under-diagnosed, with an average of more than two years of repeated clinical exams and three different physicians before women are diagnosed and treated [1].

## 2. PCOS and Its Potential Origins

PCOS is strongly familial [14,15,16] and highly heritable [17], with ~60% of daughters born to women with PCOS manifesting their own PCOS phenotype during adolescence and young adulthood [18]. Mounting evidence suggests, however, that heritable defects leading to ovarian hyperandrogenism contribute to the manifestation of PCOS [19]. Significant clinical sequelae emerge at puberty, including acne, infertility, endometrial hyperplasia, obesity, T2D, sleep apnea, cardiovascular disease, mood-affective disorders, and sexual dysfunction [1,3,7,20,21]. PCOS is a uniquely challenging, multi-faceted disorder in which progressive obesity enhances severity of phenotype while diminishing wellbeing and quality of life [22]. The findings that hyperandrogenism and metabolic dysfunction cluster in PCOS families [23,24] are consistent with genetic susceptibility. Other causes of hyperandrogenism may also contribute to the development or aggravation of PCOS, including LH hypersecretion, adipogenic constraint and obesity leading to compensatory hyperinsulinemia arising from insulin resistance, as well as fetal androgen exposure [3,25,26]. Hyperandrogenism in PCOS may thus ultimately occur as vicious functional synergism among several genetic, developmental and pathophysiological variables. Regardless of its origins, hyperandrogenism in females may serve as the final common pathway mediating the development of PCOS phenotypes [3,11,27]. Such etiopathogenic mechanism(s) also include the non-hyperandrogenic adult PCOS phenotype (type D), which shares gene variant associations with hyperandrogenic phenotypes [28]. Moreover, gestational T excess produces female macaque offspring that exhibit both hyperandrogenic and non-hyperandrogenic adult phenotypes [12], while hyperandrogneic and non-hyperandrogenic PCOS-related phenotypes also occur naturally in female macaques [13].

PCOS signs, however, are found before puberty. Infants born to mothers with PCOS, and thus likely to exhibit PCOS themselves [17,18], demonstrate elongated anogenital distance [29] and facial sebum [30], both indicative of gestational exposure to T; they also have elevated circulating levels of ovarian antimullerian hormone (AMH) [31,32], indicative of exaggerated antral follicle numbers typical of polycystic ovaries. Peripubertal daughters of PCOS women similarly exhibit elevated AMH levels [18,33], in addition to increased circulating LH levels [18], potentially from accelerated hypothalamic gonadotropin-releasing hormone (GnRH) pulsatile release (a trait programmed in rhesus macaques by gestational T exposure or prepubertal androgen excess [34,35]), and increased proclivity for synthesizing the highly biopotent androgen, dihydrotestosterone (DHT) [36], suggesting enhanced androgen action within target tissues. Not surprisingly, therefore, a major goal of pediatric medicine includes identifying girls at risk of PCOS from reliable prepubertal characteristics [37], in order to initiate early preventive therapy and/or treatment [37,38].

## 3. The Evidence for Genetic Origins of PCOS

A number of PCOS candidate genes regulating gonadotropin and ovarian function are proposed as enabling ovarian hyperandrogenism. They were identified from family-based and extensive genome-wide association studies (GWAS), as well as rare gene variant association testing (whole genome sequencing) [14,15,16,24,28]. While suggesting that future PCOS risk assessments for women may be possible based on an individual’s genotype, and may lead to tailored clinical management, currently identified putative PCOS risk genes account for <10% of PCOS heritability [24]. At least 26 replicated PCOS risk genes have emerged from studies of human populations [24,28,39,40,41,42], regulating a variety of reproductive functions, including gonadotropin secretion *(FSHB),* gonadotropin action and ovarian function *(AMH* and *AMHR2; LHCGR, STON1* and *GTF2A1L; FSHR; DENND1A; RAB5B* and *SUOX: HMGA2; C9orf3; YAP1; TOX3; RAD50; FBN3)* as well as metabolic *(THADA, GATA4* and *NEIL2, ERBB2, ERBB3, ERBB4, SUMO1P1, INSR, KRR1)* and neural *(KCNA4)* function. In particular, the post-transcription truncated isoform of *DENND1A* (DENND1A.V2) is over-expressed in women with PCOS and is functionally implicated in ovarian theca cell hyperandrogenism [43]. In addition, as might be expected from pronounced metabolic dysfunction in most PCOS cases, mothers and fathers of women with PCOS have increased prevalence of T2D, metabolic syndrome and dyslipidemia [44,45,46,47]. With the exception of *THADA* and *INSR,* however, PCOS risk genes do not associate with T2D or obesity [24]. Since currently identified PCOS risk genes account for so little of PCOS prevalence [16,24], etiopathogenesis is considered a combination of polygenic, epigenetic and developmental contributions [7,48,49], exaggerated by obesity or ameliorated by lifestyle [50,51].

## 4. The Evidence for Developmental Origins of PCOS from Clinical Studies

In considering developmental origins for PCOS, maternal–fetal environmental modification of the fetal female epigenome contributes to its transgenerational transmission [52,53,54] and can provide an additional mechanism beyond inheritance of gene variants to PCOS-like trait heritability. Amniotic fluid from daughters of women with PCOS exhibit male-similar T levels during mid-gestation, exceeding levels in mid-gestation daughters of women without PCOS [55]. As mid-gestation amniotic fluid T originates from the fetus [56,57], elevated T levels suggest hyperandrogenism in fetal daughters of women with PCOS during a crucial developmental window when female NHPs and humans are vulnerable to PCOS-like developmental programming [34,58]. Consistent with these findings and the well-established, androgen receptor-mediated, elongation of the anogenital distance (AGD) as an initial component of genital virilization, newborn daughters of women with PCOS [29], as well as adult PCOS women [59,60,61], exhibit elongated AGDs. Differential patterns of DNA methylation in newborn girls of PCOS women [52], as well as in adult PCOS women themselves [26,62,63], implicate epigenetic modifications during a critical developmental window, potentially indicative of changes in degree of individual gene expression.

In addition to such evidence for gestational hyperandrogenism contributing to PCOS etiopathogenesis, gestationally diabetic in utero environments [64,65,66,67], as well as poor intrauterine nutrition and fetal growth restriction [67,68,69], contribute developmental, likely epigenetic [37,70,71], programming to women with PCOS. Human placentae readily convey maternal glucose to the fetus engaging a progressively maturing fetal pancreatic beta cell response, but preventing transfer of maternal insulin [72]. While the 40% incidence of gestational diabetes in women with PCOS may be driven more by pre-conception BMI and lifestyle than PCOS per se [73,74], such metabolically challenged pregnancies contribute not only to fetal female hyperglycemia [66,75], but may also contribute to fetal female hyperandrogenism through diminished placental aromatase [76].

## 5. Attributes of Indian Female Rhesus Macaques Enhance Their Use in Clinical Translational Research, with Particular Relevance to PCOS

Progress towards prevention or cure for PCOS, however, has been hindered by evolving diagnostic criteria, underappreciation of pre-PCOS characteristics manifest during infancy or childhood, a defining mechanistic pathogenesis, as well as the historic lack of readily available, naturally occurring or experimentally induced animal models encompassing the complexity of PCOS and its multiple phenotypes. In this review, we will thus focus on the specific contributions made by nonhuman primate, Indian rhesus macaque (*Macaca mulatta*) models to improve our understanding of PCOS etiopathogenesis, antecedents to onset of PCOS during adolescence, naturally occurring PCOS-like phenotypes, transgenerational transmission of traits, and the promise of genetically based manipulations to reveal novel therapeutic targets.

Translating research insight into the clinic for a complex disorder such as PCOS requires robust genomic and phenotypic animal models enabling development and testing of therapeutic approaches with high likelihood of translational success. In this regard, female Indian rhesus macaques share a considerable degree of genomic, developmental, physiological, anatomical, neurological, behavioral and aging similarities to humans. The Indian rhesus macaque is the most widely employed nonhuman primate in translational research, and for which there is the most information concerning genome structure and expression. It shares a close evolutionary history with humans, as evidenced by the strikingly similar breadth of natural disease susceptibility [77,78], including PCOS [13,79]. Comprehensive genome structure and gene expression data for Indian rhesus macaques (97.5% DNA sequence identity with humans in protein-coding exons, 93% sequence identity overall) confirm a close evolutionary history with humans. Previous studies of both single gene disorders and complex polygenic diseases demonstrate that damaging mutations in rhesus macaques often generate pathological phenotypes that are highly similar, if not indistinguishable, from the analogous diseases in humans [78,80,81,82,83,84,85] (Table 2). In development of these novel macaque models of human genetic disease, US laboratory rhesus macaques demonstrate more than twice the genetic variation of human populations [86], thus providing an outstanding opportunity to identify functionally significant genetic mutations. There is thus a strong likelihood for identified gene variants in the rhesus macaque exome, within previously identified human PCOS candidate genes, to have comparable functional consequences to those found in humans [16,43].

In addition, compelling parallels in biological processes and functions shared by female rhesus macaques and humans, further enhance this translation relevance. Notably during their reproductive years, adult female Indian rhesus macaques exhibit approximately monthly menstrual cycles, a fundamental reproductive trait shared with women, but with few non-primate mammals [87,88]. Female rhesus macaques exhibit a distinct follicular phase, during which pituitary FSH-driven ovarian follicular development supports selection of a single dominant, 5–7 mm diameter follicle from a cohort of non-dominant antral follicles, leading to LH-surge driven ovulation of a single oocyte-cumulus complex at mid-cycle [87]. In many non-primate mammals, however, ovarian follicle cohort development commences during the previous luteal phase, commonly with maturation of multiple dominant follicles during a relatively truncated follicular phase [87]. Thus, in female macaques and humans, GnRH and LH elude luteal suppression during dominant follicle selection, enabling hypergonadotropic perturbation.

At the neuroendocrine control level of ovarian function, macaque anterior pituitary gonadotrope cells are partially emancipated from hypothalamic determination of function. Primate pituitary gonadotropes, unlike those of non-primates, can coordinate all negative and positive feedback responses of gonadotropin to ovarian follicle hormone release provided they receive unvarying, but physiologically appropriate, GnRH episodic release [89,90]. Such devolution of function is consistent with primate hypothalamic reproductive neuroendocrine control residing more within the mediobasal hypothalamus, alone [91,92], thus providing fundamental regulatory differences to female non-primates.

Progressing to ovulation, it is spontaneous in macaques, as in women, and attributes of the macaque corpus luteum (CL) are atypical of female non-primates [87]. In macaques and humans, the single corpus luteum (CL) releases estradiol (E_2_), androgens and inhibin A, in addition to high concentrations of progesterone (P_4_) and relaxin, thus extensively inhibiting gonadotropin-stimulated follicle recruitment and selection of a dominant follicle [93,94,95]. In mice and rats, in contrast, timely mating is required at the time of spontaneous ovulation in order to produce CL formation [87,95]. With regard to CL demise, in macaques and humans an endogenous biological ‘clock’ triggers intra-cellular increases in prostaglandin concentrations that diminish luteal cell responses to LH stimulation [96,97]. These changes within the CL dictate its regression after ~12–16 days, and the demise of P_4_-supported, highly vascular endometrial tissue, leading to onset of menses. In contrast to sheep and cattle, there is no requirement for a uterine-secreted CL inhibitor [98,99]. If fertilization of the ovulated oocyte occurs, implantation of the subsequent embryo leads to maternal pregnancy recognition, including ‘rescue’ of the CL from demise by fetal trophoblast release of chorionic gonadotropin (CG) that binds to LH/CG receptors on the ovarian CL. The rapid development of the highly invasive, villous hemochorial placenta subsequently succeeds the CL as the major generator of P_4_ and also provides exceptional capacities (compared to non-primates) to aromatize, conjugate and inactivate maternal androgens, thus buffering female primate fetuses from maternal androgen excess unless concentrations equivalent to those of a mature testis are realized [100]. Interestingly, compromised morphology and function of the PCOS placenta in human pregnancies [67,101,102] may compromise its androgen-diminishing qualities, potentially leaving PCOS female gestations exposed to PCOS maternal androgen excess [103]. Taken together, these shared post-ovulatory and post-conception reproductive attributes between female rhesus macaques and women strike clear contrasts to the differentially regulated CLs and less aggressive placentae of female non-primates [87].

We also need to briefly consider prepubertal females and primate-typical suppression of mature ovarian function since the mechanism(s) differ fundamentally from non-primates. This is of considerable importance since identifying pre-PCOS traits before adolescence would enable early intervention and diminish or delay onset of PCOS traits [104]. Following “mini puberty” of infancy in female macaques and girls, a neurally based mechanism, independent of circulating ovarian E_2_, inhibits hypothalamic-pituitary-ovarian (HPO) function until onset of puberty [105,106]. In non-primates, however, very low levels of E_2_ secreted from immature ovaries are essential for maintenance of HPO inhibition until chronological age-typical onset of puberty [107]. In non-primate females with PCOS-like traits, therefore, perturbed ovarian and hypothalamic function can both contribute to perturbed prepubertal development, whereas in female macaques and girls, only altered hypothalamic regulation perturbs prepubertal development. In addition, there is an adrenal prepubertal androgenic contrast between female mice and rats versus female primates: adrenarche or the onset of adrenocortical androgen biosynthesis. In rats and mice, there is no postnatal development of an androgenic zona reticularis in the adrenal cortex [108], and thus adrenal androgens do not contribute to PCOS-relevant pathogenesis in these rodents. In macaques, androgenic zona reticularis development occurs during early infancy, whereas in apes and humans it occurs in juveniles prior to puberty [109].

## 6. Female Indian Macaque Models of PCOS

**Figure 1:**
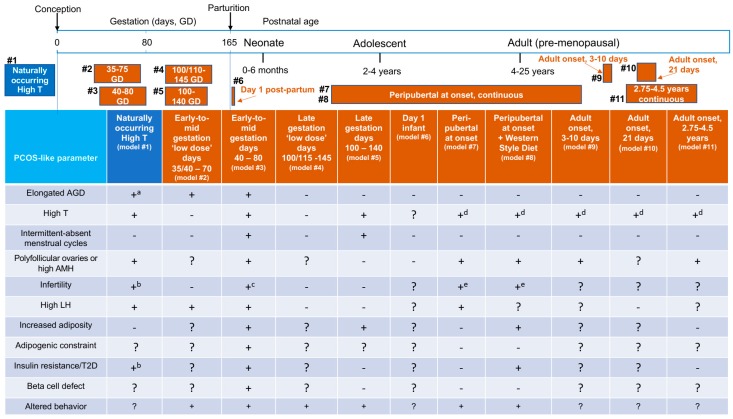
Developmental chronology of experimental manipulations utilized to generate female Indian macaque models for polycystic ovary syndrome (PCOS) and induced phenotypic traits in comparison to naturally occurring hyperandrogenic females. a, positive correlation between circulating T and AGD in High T females, alone; b, only in High T females with circulating T ≥ 2 SD above population mean T; c, <20% of fertilized oocytes reach blastocyst in vitro; d, high circulating T produced by continuous exogenous treatment; e, poor embryo quality contributing to pregnancy failure. References: model #1 [13], model #2 [111], model #3 [100,112], model #4 [111], model #5 [100,112], model #6 [113], model #7 [35,114], model #8 [35,114], model #9 [115,116], model #10 [117], model #11 [118,119,120].

Selected PCOS-like phenotypic traits of Indian female rhesus macaque models are described in Figure 1, alongside either naturally occurring origins or experimentally induced derivation (Table 3), and provide developmental context for chronological female age at the time of androgen exposure. Combining all PCOS-like traits of all macaque models together in Table 4, illustrates the compelling female macaque mimicry of PCOS in women and provides overwhelming evidence of the translational relevance of macaque models. We can thus utilize fetal, infant, peripubertal, reproductive, neuroendocrine, metabolic and behavioral PCOS-related traits, as well as gestational and placental contributions to transgenerational transmission, to provide unique insight into PCOS etiopathogenesis.

Experimentally induced, early-to-mid gestation T exposure (model #3, Figure 1, Table 3) generates the most comprehensive reproductive, neuroendocrine and metabolic phenotypic mimic of PCOS, followed closely by naturally occurring hyperandrogenism (with hypothetical mid-gestation onset of hyperandrogenism [13]), and then experimentally induced, peripubertal onset of lifetime exogenous T exposure supplemented with high-fat (Western style) diet (model #8, Figure 1, Table 3). While the remaining macaque models are less compelling, all except the infant model (model # 6, Figure 1, Table 3) exhibit at least two core tenets of a PCOS diagnosis. From this perspective, it becomes clear why macaque models are clinically recognized as providing “a paradigm shift in concepts about the pathogenesis of the disorder” since they have generated key “insight that prenatal exposure to androgens can reproduce most of the features of the human syndrome in primates” [110].

## 7. Infant and Peripubertal Reproduction-Related Endocrine and Ovarian Characteristics Preceding Adult Onset of PCOS-Like Traits

Following early-to-mid gestation T-exposure, infant female macaques exhibit hyperandrogenemia, LH hypergonadotropism and elongated anogenital distance [100,121], all preceding delayed menarche and excessive incidence of intermittent menstrual cycles during adolescence and early adulthood [122] (model #3, Figure 1). In contrast, ‘low dose’ early-to-mid gestation T (model #2, Figure 1, Table 3) only induces elongated anogenital distance and infant LH hypergonadotropism [111], suggesting that during a key gestational developmental window for androgen receptor (AR)-mediated sexual differentiation in both primates and humans [123], fetal external genitalia and neural centers regulating both the HPO axis and aspects of behavior (see below) are most responsive to programming impacts of fetal T exposure. Female external genitalia are unaffected by any of the later T exposures, while late gestation T and peripubertal T onset exposures exhibit aspects of PCOS-like reproductive or endocrine traits (Figure 1). If T administration only commences peripubertally, female macaques exhibit species-typical age at menarche (~2–2.5 years) and adolescent menstrual irregularity, but with a trend towards diminished dominant follicle selection and a transient acceleration of episodic release of pituitary LH [35]. Taken together, these macaque models suggest that pediatric detection of pre-PCOS traits in girls may be possible.

Consequently, do daughters of women with PCOS exhibit prepubertal hyperandrogenism, hypergonadotropism or peripubertal ovarian dysfunction preceding the onset of PCOS? Perhaps not surprisingly, these questions are proving difficult to answer given ethical and safety concerns of recruiting young girls into clinical research. Recently, however, carefully designed clinical studies are providing affirmative answers. Infant daughters of women with PCOS exhibit elongated anogenital distance [29], as do adult women with PCOS [59,60,61]. Prepubertal daughters of women with PCOS exhibit increased whole body 5alpha-reductase activity [36], enhancing target organ exposure to androgen action before menarche, while pre- and peripubertal daughters exhibit hyperandrogenism [18,33,124], including elevated ‘free’ T (not bound to sex hormone binding globulin, SHBG) [33], along with consistently elevated ovarian AMH levels from infancy [18,31,32] and LH hypergonadotropism during adolescence [18]. Peripubertal hyperandrogenism can persist into adulthood, associated with reduced fecundity [125], and persistent adolescent menstrual irregularities progress to a PCOS diagnosis in ~57% of cases [126]. The latter emulates the ~60% diagnostic progression expected from a separate longitudinal study of PCOS families following newborn daughters into adulthood [18]. With regard to age at menarche, however, it is early [127], normal [128] or late [129] for women with PCOS, seemingly determined by weight during later childhood and at menarche. Overweight girls with PCOS are at greater risk for earlier age at menarche, while those who were thin before or at menarche are more likely to have a delayed menarchal age [130]. Perhaps not surprisingly in these circumstances, diagnosing adolescent PCOS currently requires girls to demonstrate hyperandrogenism and irregular menses for at least two years following menarche [37].

## 8. Adult PCOS-Related Traits in Female Macaque Models

### 8.1. Reproductive Endocrine, Ovarian, Adrenal and Infertility Traits

Female macaques with naturally occurring hyperandrogenism, as well as those exposed to early-to-mid and late gestation T, but not ‘low dose’ gestational T, all manifest endogenous hyperandrogenism as adults (Figure 1), including ovarian hyperandrogenism in both early-to-mid and late gestation T-exposed females. Only the former, however, have been shown to demonstrate additional adrenal hyperandrogenism to date [131]. In an important distinction from naturally occurring and gestational T-exposed models, macaques exposed to T starting around puberty or in adulthood derive their hyperandrogenism from continuing exogenous administration of T and not endogenous sources.

Since there is no veterinary clinical diagnosis for PCOS, we have proposed [12,121] and refined [13] PCOS-like diagnostic criteria for female rhesus macaques from two of the following three: (1) circulating total T levels ≥1 SD above the population or control mean T level (≥0.31 ng/mL, 1.1 nmol/L), (2) menstrual cycle intervals ≥34 days, (3) ≥10, ~1+ mm diameter antral follicles in at least one backlit, translucent ovary illuminated during laparoscopy or circulating AMH levels ≥10 ng/mL. Ultrasonography is insufficiently refined to reliably identify unstimulated macaque ovaries and individual 0.5 mm diameter antral follicles. Nevertheless, such close approximation to PCOS criteria for women highlights closely shared reproductive and endocrine attributes. Utilizing both human and macaque criteria to assess incidence of phenotypes (types A–D), Table 1 illustrates a majority of classic (type A and B) PCOS and PCOS-like phenotypes, respectively, in both clinical referral studies of women with PCOS and in macaques exposed to T during gestation (models #3 and #5, Figure 1). This bias towards classic PCOS stands in contrast to the mostly non-classic type C and D phenotypes exhibited by PCOS women selected from local human populations and naturally hyperandrogenic female macaques (model #1, Figure 1). Considering these differences in predominance of PCOS phenotypes, it is interesting to speculate whether gestational T exposure plays a crucial developmental role in programming classic PCOS phenotypes with impaired hypothalamic GnRH regulation and dysfunctional ovarian follicle development, as well as adipogenic constraint leading to lipotoxicity and insulin resistance (see below). The PCOS phenotype bias in clinical referral studies might reflect a selected population enriched for subjects with developmental etiopathogenic origins for PCOS who might therefore benefit from early lifestyle or therapeutic intervention prior to conception or following birth and before the onset of puberty, such as weight loss, insulin sensitizer, anti-androgen or a combination therapy approach.

Interestingly, PCOS-like intermittent or absent menstrual cycles are only observed in early-to-mid and late gestation T-exposed females (models #3 and #5, Figure 1). Without irregular menstrual cyclicity, the remaining female macaque groups with hyperandrogenism thus exhibit a PCOS-like phenotype reminiscent of the ovulatory or type C PCOS phenotype in women. Peripubertal onset T exposure in female macaques supplemented with a high fat diet (T + WSD), however, does lead to luteal insufficiency [35], suggesting impaired dominant follicle maturation prior to ovulation and a potential for subsequent cycle disruption. These changes are associated with diminished ovarian vascular perfusion likely compromising function of both the preovulatory dominant follicle and the subsequent CL [114]. Peripubertal onset of high fat/calorie diet, alone however, fails to induce endogenous hyperandrogenism or polycystic ovaries in female macaques [114], suggesting that obesity, alone, is not etiopathogenic for PCOS.

While irregular menstrual cycles are limited to gestational T exposed female macaques, infertility is not. Most females with extreme natural-occurring hyperandrogenemia fail to attain pregnancy despite ample mating opportunities [13], while peripubertal T onset delays attainment of pregnancy [132]. Mild uterine endometrial P_4_ resistance demonstrated by peripubertal T+WSD onset (model #8, Figure 1) may additionally contribute to pregnancy delay and pregnancy loss [132]. In this regard, positive associations between uterine endometrial thickness in naturally hyperandrogenic females and circulating insulin, HOMA-IR and waist-to-hip ratio [13], may further implicate synergistic hyperandrogenic and metabolic contributions to pregnancy disruption that are manifest in peripubertal T-onset macaque models [132], and could resemble pregnancy complications in women with PCOS [67,101,102]. Impaired oocyte quality may provide additional underlying mechanism(s) diminishing fertility and fecundity in women with PCOS [133,134], as emulated by female macaques exposed to early-to-mid gestation T [135], as well as peripubertal onset of T [132], exhibiting poor embryo quality with diminished embryonic progression to morula/blastocyst stages, likely contributing to pregnancy failure or delayed attainment of pregnancy. In addition, females exposed to early-to-mid gestation T exhibit diminished oocyte developmental competence and fertilization rates [135], further reducing likelihood of pregnancy. These macaque findings would suggest that women with PCOS, who are seeking infertility treatment, are highly likely to benefit from pre-conception lifestyle, therapeutic or combination therapies in terms of diminished pregnancy complications and healthier babies.

### 8.2. Neuroendocrine PCOS-Related Traits in Macaque Models

Neuroendocrine hypothalamic dysfunction, long identified with PCOS in women [136], includes diminished hypothalamic responsiveness to the actions of E_2_ and P_4_ [137,138]. Altered hypothalamic response to E_2_ has relevance to perturbed metabolic function in hyperandrogenic females since rodent models have shown that T diminishes E_2_ action in the female hypothalamus [139], while estrogen receptor alpha (ERα)-expressing neurons in the ventromedial nucleus of the hypothalamus transduce the stimulatory effects of E_2_ on energy expenditure and body weight regulation [140,141,142,143]. Current studies in adult female macaques are discerning whether or not diminished hypothalamic expression of ERα or of the estrogen-synthesizing enzyme, aromatase (*CYP19A1*) emulate the low energy, hyper-adipose and hypergonadotropic female phenotype reminiscent of women with PCOS and mouse genetic model counterparts.

When considering hypothalamic regulation of female reproduction, early-to-mid gestation T exposure induces the most comprehensive and compelling mimic of PCOS reproductive neuroendocrine dysfunction with increased episodic release of LH [34], and likely hypothalamic GnRH, increased pituitary LH responsiveness to exogenous GnRH [144], chronically elevated basal LH during both follicular and luteal phases of the menstrual cycle [112] as well as during anovulatory periods, with underlying failures of E_2_- and P_4_-mediated LH negative feedback [34]. Compromised steroid hormone actions implicate impaired ERα expression and/or action in hypothalamic neuropeptide regulation of GnRH episodic release, including ERα expressing kisspeptin and GABA neurons [145]. Peripubertal onset of T exposure transiently induces accelerated episodic release of LH in late adolescence, but this is lost in early adulthood when only follicular phase elevation of basal LH remains [35] in otherwise regularly cycling T-exposed females. In early-to-mid gestation T-exposed female macaques, persistent impairments in E_2_- and P_4_-mediated negative feedback regulation of hypothalamic LH, and likely GnRH, probably contribute to their more resilient PCOS-like neuroendocrine phenotype. In fact, it is precisely such faithful recapitulation of PCOS-typical diminished neuroendocrine action of E_2_ and P_4_ that makes this particular macaque model (model #3, Figure 1) so compelling as an etiopathogenic mimic of PCOS in women.

Most women with PCOS exhibit elevated basal LH levels due to accelerated episodic release of pituitary LH [136], and thus likely hypothalamic GnRH, increased pituitary LH responsiveness to GnRH [146,147], and diminished negative feedback efficacy of both E_2_ and P_4_ [137,138], with such neuroendocrine abnormalities arising during adolescence [18,139]. Collectively, the translational insight gained from macaque models (Figure 1) suggests that PCOS reproductive neuroendocrine pathogenesis occurs during an early-to-mid gestation developmental window and that adolescent onset of exogenous hyperandrogenism can elicit a component of the hypergonadotropic phenotype. Adolescent onset of androgen-diminishing approaches, including therapeutic and/or lifestyle interventions, could therefore prove effective in ameliorating or preventing hypothalamic programming.

LH hypergonadotropism is also reported for naturally hyperandrogenic female macaques (model #1, Figure 1). With accompanying elevated LH:FSH ratio, the neuroendocrine defect is reminiscent of relative FSH deficiency and LH excess found in women with PCOS [148]. Such relative FSH deficiency likely contributes to anovulation in women with PCOS, since only low doses of FSH are required for ovulation induction [149] or controlled ovarian stimulation for in vitro fertilization [150].

Adult onset T-exposed female macaques (models #9–11, Figure 1), in contrast, do not exhibit LH hypergonadotropism, as exemplified by an ovary-intact macaque model in which endogenous GnRH-mediated LH and FSH release is inhibited by GnRH receptor antagonist administration followed by episodic (hourly) IV infusion of recombinant human LH and FSH in females accompanied by 15 days of exogenous T or saline SC administration (model #10, Figure 1, Table 3). In this model, exogenous T fails to amplify LH levels, ovarian E_2_ release or ovarian weight above values achieved in control females [117]. Taken together, these neuroendocrine findings suggest both early-to-mid gestation and peripubertal developmental windows of susceptibility to T-induced PCOS-like hypergonadotropism. The hypergonadotropism of peripubertal T onset models, however, succumbs to mature ovarian hormone regulation [35], in contrast to early-to-mid gestation T exposure models, suggesting potential absence of or more transience in hypothalamic neuronal reprogramming in peripubertal T-onset models.

### 8.3. Metabolic PCOS-Related Traits in Macaque Models

Women with PCOS demonstrate diminished lipid oxidation [151] as a component of metabolic inflexibility [152], and exhibit lower lipid oxidation-dependent thermogenesis [153], all contributing to increased obesity, adipogenic constraint-enhanced hyperlipidemia and insulin resistance [154], pancreatic beta cell decompensation and T2D [155]. Enhanced adrenergic (sympathetic nervous system noradrenalin) stimulation of visceral fat lipolysis compounds a hepatic hyperlipidemic environment [156]. When considering macaque T treatments or natural hyperandrogenemia without supplementation with high fat or “Western Style” diet, gestational T-exposed female macaques, alone, exhibit increased adiposity accompanying PCOS-like reproductive traits (models #3 and #5, Figure 1). Early-to-mid gestation T-exposed females (model #3) exhibit increased visceral adiposity [157], or ‘metabolic obesity’, whereas late gestation T-exposed females (model #5) exhibit whole body increases in adiposity without preferential visceral accumulation [158] or glucoregulatory defects [159]. Preferential visceral fat accumulation in early-to-mid gestation T-exposed females likely arises from endogenous hyperandrogenism inhibiting gene expression of a key adipogenic transcription factor, C/EBPalpha [160], thus constraining SC adipocyte maturation and safe lipid storage, while enabling increased lipid accumulation in more pro-lipolytic visceral adipocytes [154,156]. Such AR-mediated distortion of lipid accumulation [26] likely enables hyperlipidemia-associated insulin resistance [154] and pancreatic beta cell compensation, ultimately compromising islet integrity and glucose homeostasis with inevitable progression to T2D [161]. The absence of such lipotoxic progression in late gestation T-exposed female macaques in adulthood may relate to closure of an early-to-mid gestation developmental window for programming of adipose function.

Partially emulating the metabolic outcomes of early-to-mid gestation T-exposure, female macaques exposed to peripubertal onset of T, and supplemented with a high fat diet (T + WSD), demonstrate increased abdominal ‘android’ fat and abdominal circumference indicative of increased visceral adiposity [132]. In peripubertal T onset females, diminished basal lipolysis in both SC and visceral abdominal fat depots co-occurs with augmented insulin-mediated FFA uptake into visceral adipocytes, alone, contributing to enlarged visceral, but not SC, adipocytes [162]. Since adrenergic (sympathetic nervous system noradrenalin) stimulation of lipolysis is also diminished only in SC adipocytes, unaffected adrenergic stimulation of lipolysis in enlarged visceral adipocytes likely contributes increased lipid release into the liver, with subsequent adiposity-associated insulin resistance and compensatory hyperinsulinemia [162]. T + WSD female macaques thus demonstrate the need for the onset of both hyperandrogenism and high fat diet during adolescence to evoke the adult metabolic derangements engaged by early-to-mid gestation T exposure, alone (models #8 vs. #3, respectively, Figure 1). Female macaques receiving peripubertal T onset without high fat supplementation do not exhibit such metabolic dysfunction and weight gain [132,162]. Since blood lipid levels, pancreatic beta cell defects and diminished adipocyte size are not reported, it can only be speculated that neither adipogenic constraint nor lipotoxicity become a pathological consequence when female macaques are exposed to peripubertal onset of hyperandrogenism, alone. This may provide a valuable translational insight for young women with PCOS: prevent or counteract diet-enabled weight gain, and any remaining hyperandrogenism will pose little metabolic risk.

### 8.4. Behavioral PCOS-Related Traits in Macaque Models

Sexual dysfunction [163] and depression [164] are increasingly coincident sequelae in women with PCOS [21]. Four macaque models of PCOS are potentially of relevance to their developmental origins. Both early-to-mid and late gestation T-exposures reprogram (“organize”) prepubertal and adult female macaque behavior (models #2–5, Figure 1). While they may best inform behavioral issues in women with classic congential adrenal hyperplasia (CAH), whose early gestational hypocortisolemia unleashes fetal adrenal hyperandrogenism [165], the implication of mid-gestational T exposure in women with PCOS from findings of amniotic fluid hyperandrogenism and increased anogenital distance in newborn daughters, also suggests macaque model relevance.

Early-to-mid or late gestation ‘low dose’ T-exposed female macaques exhibit increased aspects of male-typical infant vocalizations [166,167], while behavioral responses of their mothers remain typical of those displayed to daughters, but not to sons [166]. As juveniles, higher dose gestational T-exposed females (models #3 and #5, Figure 1) exhibit increased male-like mounting of juvenile peers, and late gestation T exposure additionally contributes increased male-like rough play [168]. When adult, early-to-mid, but not late, gestation T-exposed female macaques display infrequent sexual initiation to males [169] and, when administered T, display increased male-like sexual solicitation, sexual contacts and mounting of females [170], atypical of adult females macaques [168]. In addition, both peripubertal T-onset macaque models, while able to spontaneously conceive on mating with males in adulthood and rear offspring [132], exhibit diminished locomotion activity likely contributing to their increased adiposity when fed a high fat diet [171]. Peripubertal T-onset female sexual behavior, however, has not been systematically examined.

Translating these “organized” behavioral findings to women with PCOS is far from straightforward. For example, in prepubertal 46, XX girls with classic congenital adrenal hyperplasia (CCAH), with known T exposure from early gestation, rough play and preferences for boy-typical toys are increased [58]. When adult, women with CCAH experience increased sexual arousal to women [58] together with increased sexual dysfunction [172], while retaining female gender identity [172]. 46,XY women lacking bioeffective androgen receptors (CAIS), in contrast, are born with intra-abdominal testes, are indistinguishable from 46,XX girls and women in terms of body habitus (except for absence of body hair), as well as female gender identify and sexual orientation [173], and exhibit only one minor difference in fMRI-assessed neural responses to sexually arousing images [174]. The fMRI-related difference, however, is not male-like. CAIS women, nevertheless, do experience increased sexual dysfunction potentially related to challenging clinical management, including testicular removal due to cancer risk, hormone replacement therapy and vaginal reconstruction [175]. Of additional relevance here, particularly in terms of rodent-primate model differences, local brain aromatization of T to E_2_ is required to “organize” subsequent male-like or male-typical juvenile and adult behavioral responses in rodents [176], but not in primates [168]. Together with the gene variant-based CCAH and CAIS examples, above, and that the non-aromatizable androgen, dihydrotestosterone (DHT) faithfully replicates T-induced behavioral organization in both gestational T-exposed macaque models for PCOS [168], models #3 and #5 (Figure 1), it is AR- and not ER-mediated brain action that “organizes” primate behavior. 

T-mediated behavioral reprogramming, if emulated in women with PCOS, may therefore pose challenges for traditional female gender roles in human societies, potentially leading to sexual dysfunction and depression. In addition, diminished locomotion, if similar to that found in peripubertal macaque models, will compound depression [177] and weight gain in women with PCOS. Recent reports from *in utero* androgen excess rodent models clearly demonstrate anxiety-like behavior in female offspring accompanied by the upregulation of amygdala gene expression, including corticotropin-releasing hormone and AR [178], a neural site and neuropeptide system implicated in the pathogenesis of anxious phenotype in macaques and humans [81], leading to depression.

### 8.5. Gestational and Placental Contributions to Transgenerational Transmission of PCOS-Like Traits

Gestational diabetes is one among many complications of pregnancy experienced by women with PCOS related to gestational weigh gain [74]. Since comparable compromised gestational findings are emerging following experimentally induced hyperandrogenism in female macaques (models #3, #5, #7, #8, Figure 1), and are enhanced by increased maternal body fat and gestational weight gain (models #3 and #8, Figure 1), maternal T excess in combination with increased maternal adiposity may compromise gestation in primates, including humans. Impaired placental syncytiotrophoblast villous maturation accompanied by diminished placental vascularity in both hyperandrogenic female macaques [132] and women with PCOS [101] may contribute to fetal hypoxia, impaired fetal development and diminished fetal circulating levels of non-esterified free fatty acids [75]. While female fetal head size [75], and relative abdominal circumference and body weight [132], are all increased during hyperandrogenic macaque pregnancies, newborn female birthweights are normal. Subsequent infant growth is increased, however, in the context of female newborn hypoglycemia and infant hyperinsulinemia [75], all parameters typical, in humans, of female offspring born following diabetic gestation that bestows increased risk of adult obesity and T2D [65,66]. Gestational T excess induction of metabolically compromised gestation may thus provide the developmental origins necessary for metabolic pathophysiology accompanying PCOS. Pre-conception lifestyle and therapeutic approaches to maternal weight reduction and improved glucoregulation can ameliorate the metabolic environment of subsequent PCOS pregnancies [179], but gestation-based therapies, including maternal metformin administration, risk increased weight gain and accelerated glucoregulatory dysfunction among offspring beyond that achieved by PCOS alone [180]. Rhesus macaques are especially suited for gestation-based research, accelerating translation-ready therapies beyond any non-primate or human based approaches. Taken together, these findings suggest that interventions aimed at diminishing maternal body weight and glucoregulatory function prior to conception would diminish metabolic developmental programming of girls born to women with PCOS and of female infants born to PCOS-like macaque mothers.

### 8.6. Translational Considerations

PCOS-like phenotypes in female Indian macaques arise from diverse and complex pathogenic origins that include contributions from androgenic and metabolic perturbations of the intrauterine environment that may well co-occur in PCOS gestations. Their convergence in naturally hyperandrogenic and experimentally induced hyperandrogenic female rhesus macaques strongly supports a pathogenic concept of combined genetic, epigenetic and developmental origins for PCOS among ancestral primates. Translating understanding of PCOS pathogenesis from rhesus macaque models into novel clinical intervention is, however, not without its challenge. For example, if anti-androgen therapy such as flutamide were to be administered to pregnant mothers in order to prevent fetal T programming of PCOS traits in female offspring, the anti-androgen therapy may induce flutamide-specific behavioral programming. Certainly, when administered to rhesus macaque dams, flutamide-exposed female offspring exhibit masculinized vocalizations as infants [166], and in adulthood display cognitive dysfunction [181] and diminished interest in infants [168]. Without reliable predictions of gestational age at onset of increased T exposure, or understanding of the degree or duration of T excess in human pregnancies carrying fetal fetuses at risk for PCOS after birth, the risk of detrimental postnatal outcomes, such as those reported for gestational flutamide-exposed female macaques [168], outweigh potential benefits from gestational therapy aimed at ameliorating PCOS traits. 

Gestational application of the insulin sensitizer, metformin, based on initial promising results suggesting abrogation of PCOS-related metabolic dysfunction [182,183] has highlighted the unintended consequences of premature gestational intervention. In a randomized clinical trial of daily metformin or placebo given to pregnant PCOS women from at least 12 weeks of gestation, newborn girls exposed to metformin during gestation had increased head size [184], followed by subsequent increased adiposity and insulin resistance by 4 years of age compared to age similar control peers who were also born to women with PCOS [185]. In other words, gestational exposure to metformin enhanced transgenerational transmission of metabolic dysfunction [72], rather than diminishing it.

A more effective translation of rhesus macaque model findings into improved clinical practice may involve identifying girls at risk for PCOS and initiating lifestyle intervention strategies before puberty. Since reliable, and safely accomplished, fetal indicators of androgen excess are beyond current clinical capability, neonatal or infant biomarkers such as increased measures of sebum content in forehead wipes [30], anogenital distance [29] or circulating AMH levels [31], may become sufficiently refined to provide specific and sensitive identifiers of PCOS risk. Early interventions ameliorating onset or degree of PCOS traits would hold promise for diminishing PCOS severity. For example, 6-month treatment of adolescent hyperandrogenic girls with low-dose anti-androgen and combined insulin sensitizer (spironolactone, pioglitazone, and metformin), resulted in a 1.5–2 years delay in return of hyperandrogenism and anovulatory cycles compared to hyperandrogenic adolescents receiving oral contraceptives alone [37]. Such preventive approaches would be predicted as highly beneficial from two mutually inclusive concepts of PCOS pathogenesis derived from macaque models and clinical studies: adipogenic constraint [186] and gestational hyperandrogenism [187].

## 9. Conclusions

**Figure 2:**
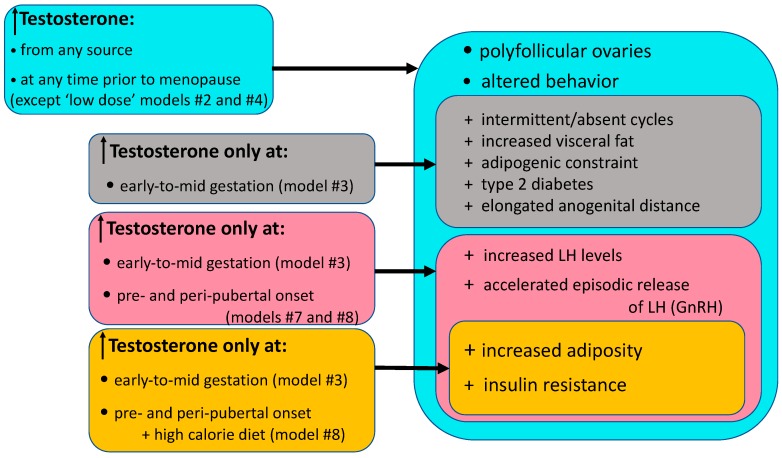
Diagrammatic illustration of commonalities in selected PCOS-like traits exhibited by female Indian macaque models.

Rhesus macaque models for PCOS provide a developmental chronology detailing specific pathophysiological outcomes induced by T excess, with or without accompanying high fat diets, occurring as early as the first trimester of pregnancy to as late as the reproductive years of adulthood (Figure 1 and Figure 2, Table 1 and Table 3). Paradigm shifts in considering mechanisms and origins of PCOS have arisen from understanding pathophysiological outcomes described in rhesus macaques models of PCOS, and have reinforced personalized clinical management depending on phenotypic presentation of patients. In addition, and unique to rhesus macaque models or PCOS, highly comparable rhesus macaque and human genomes and physiological functions enabled identification of naturally occurring PCOS-like female rhesus macaques that hold promise for genomic studies functionally linking gene variants to specific PCOS-relevant dysfunction, and therefore potentially curative interventions.

## Figures and Tables

**Table 1:** Incidence of PCOS phenotypes in women with PCOS in comparison to PCOS-like phenotypes in female rhesus macaques with PCOS-like phenotypes.

	PCOS and PCOS-Like Phenotypes ^a^			
	(% of PCOS Women and PCOS-Like Monkeys)			
	Classic Phenotypes		Non-Classic Phenotypes	
Female Population	Type A	Type B	Type C	Type D
PCOS women (clinical referrals)	49	13	14	17
	-----------62%-----------			
Female macaques				
Early-to-mid gestation T-exposed	38	25	12	25
	-----------63%-----------			
Late gestation T-exposed	20	60	20	0
	-----------80%-----------			
PCOS women (from local, unselected populations)	25	19	35	20
			-----------55%-----------	
Female macaques				
Naturally hyperandrogenic (High T)	25	8	42	25
			-----------67%-----------	

Modified from [11], with late gestation T-exposed data modified from [12]. ^a^ Phenotypes: type A, hyperandrogenism or hirsutism (Women only) (HA) + intermittent/absent cycles (OD) + polycystic ovary morphology or circulating AMH level ≥ 10 ng/mL (PCOM); type B, HA + OD; type C, HA + PCOM; type D, OD + PCOM, as described [13].

**Table 2:** Estimates of heritability (h^2^) of various complex phenotypes in US laboratory populations of Indian rhesus macaques. All heritability estimates are statistically significant at *p* < 0.02.

Phenotype	Sample Size (*n*)	h^2^	Reference
Duration of freezing behavior (inhibition)	285	0.38	[85]
Anxious temperament	238	0.36	[82]
Glucose metabolism in hippocampus	238	Right: 0.65 Left: 0.76	[82]
Infant exploratory behavior	428	0.25	[85]
Infant reaction to novel threat	428	0.24	[85]

**Table 3:** Details regarding the natural occurring or experimentally induced origins of rhesus macaque models for PCOS.

PCOS-Like Model	Naturally Occurring or Experimentally Induced	Testosterone (T) Regimen	Circulating T Levels Achieved
High T [13]	Naturally occurring (model #1)	None	≥0.31 ng/mL (1.1 nmol/L)
Gestation days [111], 35/40–75 ‘Low dose’	Experimentally induced (model #2)	T enanthate 20 mg/week, IM to dam, Early-to-mid gestation 35–40 consecutive days	?
Gestation days [100,112], 40–80	Experimentally induced (model #3)	T propionate 10–15 mg/day, SC to dam, Early-to-mid gestation 15–40 consecutive days	~0.30 ng/mL (1.1 nmol/L)
Gestation days [111], 100/110–145 ‘Low dose’	Experimentally induced (model #4)	T enanthate 20 mg/week, IM to dam, Late gestation 35–45 consecutive days	?
Gestation days [112], 110–140	Experimentally induced (model #5)	T propionate 10 mg/day, SC to dam, Late gestation 25–30 consecutive days	?
Postpartum day 1 [113]	Experimentally induced (model #6)	T 35 mg/kg, SC, Neonate 1 day	?
Onset at 1–2.5 years of age [35,114], (±Western Style diet)	Experimentally induced (models #7 and #8)	T Silastic capsules, SC, Pre/Peri pubertal, Continuous ~4 years	~1.35 ng/mL (4.7 nmol/L)
Adult onset [115,116]	Experimentally induced (model #9)	T or Dihydrotestosterone (DHT), SC, Adult 4 mg/kg T, Continuous, 3 days, 20 µg/kg T or 145 µg/kg DHT, Continuous, 5 days, 400 µg/kg T, Continuous 10 days	4 mg/kg T: ~31 ng/mL (108 nmol/L) 20 µg/kg T: ~4 ng/mL (14 nmol/L) 145 µg/kg DHT: ~6 ng/mL (21 nmol/L) 400 µg/kg T: ~13 ng/mL (45 nmol/L)
Adult onset [117]	Experimentally induced (model #10)	GnRH antagonist + exogenous LH + FSH (to control LH/FSH, ovarian hormones), Adult T or DHT, Silastic capsules, SC, 10 T capsules or 150 µg/kg DHT, DHT Continuous 21 days	30–40 ng/mL T (150 nmol/L) ~30 ng/mL DHT (70 nmol/L)
Adult onset [118,119,120]	Experimentally induced (model #11)	T or Androstenedione Adult, 10-25 mg, Silastic capsules, SC, Continuous ~1–4.5 years	0.8–1.2 ng/mL (2.8–4.2 nmol/L)

**Table 4:** PCOS traits exhibited by female Indian macaque models.

Model Traits	Model # Exhibiting Traits (# from Figure 1)
**Equivalent to diagnostic criteria**	
Rotterdam criteria	1,3,5,7–9,11
Elevated adult T levels	1,3,5,7–11
Intermittent or absent menstrual cycles	3,5
Polycystic ovaries or elevated adult AMH (≥10 ng/mL)	1,3,7–9,11
**Reproductive and endocrine traits**	
Delayed menarche	3,5
Ovarian hyperandrogenism	3
Adrenal hyperandrogenism	3
Diminished oocyte or embryo quality	3,7,8
Diminished embryo quality	3,7,8
Accelerated episodic LH release (hypothalamic GnRH)	3,7
LH hypergonadotropism	1–3,7,8
Uterine endometrial abnormalities	1,7,8
**Metabolic traits**	
Newborn hypoglycemia	3
Infant accelerated weight gain	3
Infant insulin hypersensitivity	3
Infant pancreatic beta cell over-compensation	3
Adult hyperlipidemia	3
Adult increased adiposity	3,8
Adult adipogenic constraint	3
Adult lipolytic constraint	7,8
Adult hyperinsulinemia	1,3,8
Adult insulin resistance	1,3,8
Adult pancreatic beta cell decompensation	3
Adult increased type 2 diabetes	3
**Behavioral and neural traits**	
Altered infant/juvenile behavior	2,3,5,7,8
Altered infant vocalizations	2
Increased adult sedentary behavior	7,8
Altered adult sexual behavior	3,5
**Anatomical traits**	
Elongated anogenital distance	2,3
**Gestational traits**	
Gestational hyperglycemia	3
Fetal hypolipidemia	3
Compromised placental structure and function	7,8
Altered fetal growth	3,8
